# Toward Real-Time Discharge Volume Predictions in Multisite Health Care Systems: Longitudinal Observational Study

**DOI:** 10.2196/63765

**Published:** 2025-04-30

**Authors:** Fernando Acosta-Perez, Justin Boutilier, Gabriel Zayas-Caban, Sabrina Adelaine, Frank Liao, Brian Patterson

**Affiliations:** 1 Department of Industrial and Systems Engineering University of Wisconsin–Madison Madison, WI United States; 2 UW Health: University of Wisconsin Hospitals and Clinics Madison, WI United States; 3 Department of Emergency Medicine University of Wisconsin–Madison Madison, WI United States

**Keywords:** discharge, machine learning, predict, capacity management, discharge predictions, predictive modeling, emergency department, hospital admission, hospital data

## Abstract

**Background:**

Emergency department (ED) admissions are one of the most critical decisions made in health care, with 40% of ED visits resulting in inpatient hospitalization for Medicare patients. A main challenge with the ED admissions process is the inability to move patients from the ED to an inpatient unit quickly. Identifying hospital discharge volume in advance may be valuable in helping hospitals determine capacity management mechanisms to reduce ED boarding, such as transferring low-complexity patients to neighboring hospitals. Although previous research has studied the prediction of discharges in the context of inpatient care, most of the work is on long-term predictions (ie, discharges within the next 24 to 48 hours) in single-site health care systems. In this study, we approach the problem of inpatient discharge prediction from a system-wide lens and evaluate the potential interactions between the two facilities in our partner multisite system to predict short-term discharge volume.

**Objective:**

The objective of this paper was to predict discharges from the general care units within a large tertiary teaching hospital network in the Midwest and evaluate the impact of external information from other hospitals on model performance.

**Methods:**

We conducted 2 experiments with 174,799 discharge records from 2 hospitals. In Experiment 1, we predicted the number of discharges across 2 time points (within the hour and the next 4 hours) using random forest (RF) and linear regression (LR) models. Models with access to internal hospital data (ie, system-agnostic) were compared with models with access to additional data from the other hospitals in the network (ie, system-aware). In Experiment 2, we evaluated the performance of an RF model to predict afternoon discharges (ie, 12 PM to 4 PM) 1 to 4 hours in advance.

**Results:**

In Experiment 1 and Hospital 1, RF and LR models performed equivalently, with *R*^2^ scores varying from 0.76 (hourly) to 0.89 (4 hours). In Hospital 2, the RF model performed best, with scores varying from 0.68 (hourly) to 0.84 (4 hours), while scores for LR models ranged from 0.63 to 0.80. There was no significant difference in performance between a system-aware approach and a system-agnostic one. In experiment 2, the mean absolute percentage error increased from 11% to 16% when predicting 4 hours in advance relative to zero hours in Hospital 1 and from 24% to 35% in Hospital 2.

**Conclusions:**

Short-term discharges in multisite hospital systems can be locally predicted with high accuracy, even when predicting hours in advance.

## Introduction

Care coordination and planning is a complex task for hospital systems, best illustrated by the emergency department (ED) admission process. In addition to clinical severity and needs, physicians may consider patient waiting time, level of care, or specialty fit, and bed blocking when admitting a patient from the ED. The uncertainty of future patient arrivals and discharges makes these considerations more difficult when coordinating care throughout the admission processes, potentially resulting in increased operational costs and adverse patient outcomes. For instance, an analysis performed at a large multisite hospital in Canada showed that in a period of one year, delays in admissions from the ED resulted in more than 2 million dollars in additional costs and 12.4% longer length of stays [1]. More recently, with the rise in hospital mergers and acquisitions [2], patients in the ED can potentially be admitted or transferred to different hospitals, making coordination increasingly complicated.

An effort to improve coordination and planning within and across hospitals involves centralizing decision-making. This is best illustrated by the recent emergence of capacity command centers [3-5], which are a management mechanism used to centralize care coordination workflows in the presence of a network of multiple interacting hospital facilities. With real-time centralized access to hospital data and analytics, command centers can help improve operational and patient outcomes, such as inpatient occupancy and patient delays [3-6]. They can serve as a hub to develop and manage predictive analytics models such as emergency department occupancy [7,8], inpatient intensive care unit (ICU) admission [9,10], and inpatient discharges [11-17]. In this paper, we focus on the development and evaluation of discharge prediction models. We believe such models are instrumental in improving throughput and capacity management strategies, which in modern health care systems face significant challenges.

An example of a current capacity management challenge in many hospital systems is the practice of reserving general care beds (GCBs) for incoming ICU and surgical patient transfers. Surgical patients require a postprocedural bed for safety, while ICU patients ready for transfer occupy critical ICU beds needed for new arrivals. To address this, clinicians responsible for patient placement often avoid assigning GCBs reserved for these patients to ED patients, fearing a lack of available beds later. This practice leads to ED bottlenecks and prolonged boarding times for patients needing inpatient admission. However, high-quality discharge predictions could alleviate this issue by predicting bed availability for surgical and ICU patients later in the evening, enabling decision makers to reduce the need to hold empty beds and enabling more efficient hospital capacity use.

Discharge prediction models have been previously proposed as an approach to improving operational and clinical outcomes [11-17]. However, this research has focused on making longer-term patient-level discharge predictions (ie, the next 24 to 48 hours) in single-site systems. These strategies may not provide near-term information to help bed management make immediate capacity management decisions (eg, patient placement) and do not leverage potential interactions between hospitals in a multisite system. Although few studies have explored the use of multitask learning [18] for patient flow [19,20], the area remains relatively unexplored, particularly in the context of discharge volume predictions. A recent literature review [21] identified multitask learning as a promising future research direction.

To develop and evaluate prediction models capable of predicting the number of short-term discharges (ie, in the next 1 to 4 hours) in near real time, we must first decide the granularity level we want to predict. The literature has considered 2 main types of models: patient-level models [11-15] and hospital-level models [16,17]. Hospital-level models typically use a time series approach to directly predict the number of discharges in an entire hospital or a hospital unit such as the ED [16,17]. Some patient-level models directly predict the patient’s length of stay [15], while others treat the discharge prediction problem as a classification task and predict the risk that a patient will be discharged in the next 24 to 48 hours [11-13]. We note that patient-level predictions can also be used to estimate discharge volume by aggregating the patient-level discharge probability estimates [11-13]. However, previous research has shown that aggregating patient-level forecasts can result in biased predictions [7], and constructing a dataset at the patient-hour level for multiple hospitals quickly becomes computationally challenging and complicated. Therefore, we chose to develop hospital-level models due to their greater relevancy for capacity management and ease of implementation.

Our main contribution is developing and evaluating a simple yet well-performing and computationally tractable framework to provide accurate short-term hospital-level discharge predictions in multisite health care systems. Our models were developed using more than 170,000 discharge records from 2 hospitals in our partner’s network and evaluated by 2 experiments. In the first experiment, we estimate the baseline performance of machine learning models to predict the number of discharges within the next hour and the next 4 hours at each hospital in near real time. We compared the performance of models trained using internal and external data for each hospital. In addition, we test the use of multitask learning models and benchmark against their single-task learning variants. In the second experiment, we evaluate the prediction of afternoon discharges (12 PM to 4 PM), 0 to 4 hours in advance. Based on the results of our experiments, we provide practical insights into the development and implementation of discharge volume prediction models for multisite health care systems.

## Methods

### Data

Hospital visits were analyzed using electronic health records (EHRs) from 2 hospitals in a large academic health system in the Midwest. Hospital 1 is a large-scale level 1 trauma center with 505 beds and a 58-bed ED that treats an average of 67,000 patients annually. Hospital 2 is a 56-bed hospital with a 16-bed ED that treats an average of 26,000 patients annually. Both hospitals share a common EHR instance, and retrospective data were gathered from the system. We considered all general care discharges from 8 AM to 5 PM between January 1, 2017, and August 15, 2023 (n=174,799; see Table 1 for descriptive statistics). We excluded 16,844 records that occurred between 5 PM and 8 AM since these are the hours when discharge activity is the lowest. We also excluded discharges from the intermediate, intensive, behavioral, and pediatric units to limit the scope of our study.

**Table 1:** Summary statistics.

		2017-2019		2020-2023	
		Hospital 1	Hospital 2	Hospital 1	Hospital 2
**Dependent variables**					
	Discharges, n	67,315	14,663	72,729	20,092
	Hourly discharges, mean (SD)	5.59 (4.47)	1.22 (1.47)	5.00 (4.20)	1.38 (1.74)
	4-hour discharges, mean (SD)	23.34 (12.73)	4.96 (3.95)	20.80 (12.07)	5.67 (4.93)
**Independent variables**					
	Net transfers in^a^, mean (SD)	1.63 (2.71)	0.64 (1.02)	1.43 (2.56)	0.84 (1.26)
	Discharge orders, mean (SD)	15.13 (11.04)	2.96 (2.96)	12.67 (9.59)	3.33 (3.53)
	Time in bed ≤24 hours, mean (SD)	88.87 (21.15)	13.86 (6.43)	75.05 (16.43)	16.10 (7.49)
	Time in bed ≤63 hours, mean (SD)	79.78 (15.46)	8.34 (3.69)	71.43 (13.55)	10.50 (4.04)
	Time in bed ≤148 hours, mean (SD)	86.42 (15.53)	4.45 (2.75)	82.20 (15.35)	8.22 (3.72)
	Time in bed ≥148 hours, mean (SD)	63.08 (8.93)	0.88 (1.09)	72.77 (15.45)	5.11 (3.14)
**Additional demographics**					
	Age (years), mean (SD)	58.68 (17.50)	62.88 (16.37)	60.15 (17.48)	63.44 (15.82)
	Sex (female), n (%)	34,654 (51.48)	6,550 (44.67)	37,877 (52.08)	8,467 (42.14)

^a^Net transfers in corresponds to the total balance of transfers in and out of the general care beds (GCBs) in the past hour.

### Variables

Entries in our training and testing data represent hourly time stamps from 8 AM to 5 PM. Variables were constructed for each hospital to capture the state of the system at every hour interval during the period of interest (ie, 8 AM to 5 PM). We conducted 2 experiments. In the first experiment, we consider the number of discharges within the next hour and the next 4 hours as dependent variables for every hospital. Therefore, we had 4 dependent variables: number of discharges within the next hour in Hospital 1, number of discharges within the next hour in Hospital 2, number of discharges within the next 4 hours in Hospital 1, and number of discharges within the next 4 hours in Hospital 2. In the second experiment, one dependent variable is considered for each hospital, denoting the number of afternoon discharges (ie, discharges between 12 PM and 4 PM).

The independent variables were the same for both experiments. Discharge workflows are highly dependent on time since a great number of discharges will occur from early morning to late afternoon. Thus, to capture such dependencies, we included temporal features such as the time of day, day of the week, day of the year, week of the year, and month. Similarly, due to the temporal nature of our problem, we can leverage previous patient movement patterns, such as the number of past discharges and internal transfers to and from the general care units, under the assumption that they may influence future patient movements (ie, discharges). In addition, we included independent variables related to procedural orders, such as the number of active discharge orders at the time of prediction, which are highly correlated with our dependent variables. Finally, to quantify the elapsed time a patient has spent in bed, we developed variables that categorize patients based on the time they have already spent in their beds. We defined the following categories: less than 24 hours, 24 to 63 hours, 63 to 148 hours, and more than 148 hours.

Presumably, the longer a patient has spent on a bed, the more likely it is that they will be discharged. Thus, adding such variables can help to capture such behavior at the aggregate level.

For the variables associated with past discharges, transfers, time in bed, and discharge orders, we also included the history of the 6 hours leading up to the observation point as independent variables. For example, to predict discharges in the next hour at noon, we included the number of discharges from 6 AM to 7 AM, 7 AM to 8 AM, 8 AM to 9 AM, and so forth until the period of 11 AM to 12 PM, which was the last observed period before prediction. In total, we considered 107 independent variables (see Multimedia Appendix 1 for a detailed description of each variable). In Table 1, we provide summary statistics of the study population.

### Models and Model Evaluation

We used two models: linear regression (LR) with L1 regularization [22] and a random forest (RF) [23]. For each model, we considered their single and multitask variants. Multitask variants predict all dependent variables simultaneously, whereas single-task variants only predict 1 dependent variable at a time. For the LR model, the best L1 regularization coefficient was determined by using cross-validation in the training data and testing the model with different coefficients of regularization varying from 0.001 to 0.50 in increments of 0.002. A small coefficient of 0.001 was selected. In RFs, the number of trees was chosen a priori to be 200 for both the single and multitask variants. Please see Multimedia Appendix 1 for additional details on the model tuning procedure, including Table S2 with the hyperparameters used for every trained model.

Records from August 1, 2017, to December 31, 2019, were used to train all models, and the remaining records from January 1, 2020, to August 15, 2023, were used for testing. For all experiments, out-of-sample performance was estimated using a rolling origin cross-validation approach [24]. To execute this method, we split the testing set into 10 subsets of equal size and initialized the algorithm with the training dataset (ie, all data from 2017 to 2019). In iteration 1, we train the model using the training dataset and the first subset of data from the testing dataset, and estimate out-of-sample performance in the third subset of the testing dataset. In iteration 2, the model is trained using the first subset of the training set, subsets 1 and 2 of the testing set, and evaluated using subset 3 of the testing set. This procedure is repeated until the model is evaluated in the last subset of the testing data. The out-of-sample performance in all experiments performed in this study was assessed using this cross-validation technique along with the *R*^2^ score, mean absolute error (MAE), and the mean absolute percentage error (MAPE).

### Experiment 1: Baseline Performance and System-Aware Learning

We predicted our dependent variables (ie, the number of discharges within the next hour and the next 4 hours for each hospital) at every hour interval from 8 AM to 5 PM and evaluated model performance under 3 different tiers of information: system-agnostic single-task learning, system-aware single-task learning, and multitask learning. The system-agnostic single-task (SAST) learning tier assumes that a hospital can only access its data when predicting discharges. The system-aware single-task (SAWST) learning tier assumed that hospitals could access each other’s data to make predictions. Both SAST and SAWST tiers had their associated single-task models trained for predicting each dependent variable. In the multitask (MT) learning tier, both hospitals had access to systemic data (ie, data from each other), but the models were trained using MT variants of LR and RF. In other words, the multitask learning models predicted the 4 dependent variables at the same time. The performance of the models was assessed using rolling origin cross-validation, and the metric of interest was the MAE and *R*^2^ score.

### Experiment 2: Predicting Afternoon Discharges With Time in Advance

In experiment 1, it was assumed that the models make predictions at intervals between 8 AM and 5 PM every hour. In this experiment, we fix the time to predict discharges to be between 12 PM and 4 PM and evaluate the performance of an SAWST RF model when predicting zero to four hours before 12 PM. The peak discharge period is from 12 PM to 4 PM, and early mornings are typically periods of low discharge activity. The motivation for this experiment is to consider the periods of high activity and evaluate the prediction with time in advance. The models were evaluated using rolling origin cross-validation, and the metric of interest was the MAPE. See Multimedia Appendix 1 for additional technical details on the model training procedure.

### Ethical Considerations

This study was submitted to the University of Wisconsin–Madison institutional review board (IRB; submission ID 2023-1102). The IRB determined that the proposed activity does not involve research with human subjects as defined by the Department of Health and Human Services and Food and Drug Administration regulations. Therefore, IRB review and approval were not required.

## Results

**Figure 1:**
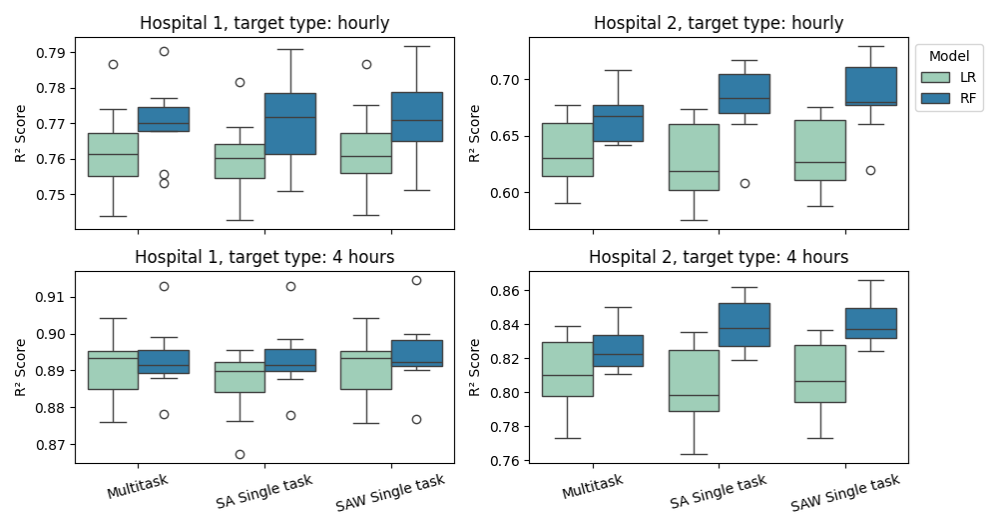
R2 score as a function of the information tier and machine learning model for all dependent variables. LR: linear regression; RF: random forest; SA: single task for system agnostic single task; SAW: single task for system aware single task.

**Figure 2:**
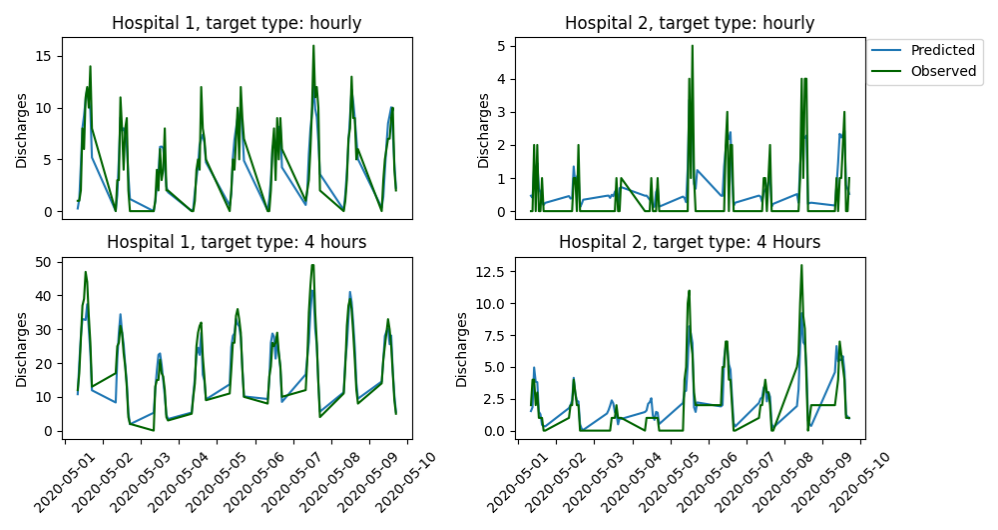
Predicted vs observed discharges for the first 10 days of the testing set.

Results for experiment 1 are depicted in Figure 1 and presented in Table 2. In addition, we provide Figure 2 as a means to observe the model performance in a visual way. In Figure 1, the box plots were constructed using the out-of-sample performance in each of the testing subsets of the rolling origin cross-validation. In Figure 2, each point on the x-axis represents an hourly time stamp, and the y-axis presents the number of discharges within 1 to 4 hours starting from that time stamp. In the case of predicting discharges within the next hour at Hospital 1, the RF and the LR models performed similarly in all information tiers, with *R*^2^ scores varying from 0.760 for the LR models to 0.771 for the RF models. The RF model outperformed the LR model for predicting discharges within the next hour at Hospital 2. In this case, the *R*^2^ score for the RF varied from 0.668 in the case of MT learning to 0.679-0.684 in the single-task tiers. The score for LR was 0.626-0.632 in the case of SAST and SAWST and 0.635 in the case of MT. All models and information tiers performed similarly for discharges within the next four hours in Hospital 1, with *R*^2^ scores varying from 0.887 (SAST LR) to 0.894 (SAST and SAWST RF). For discharges within the next four hours in Hospital 2, the SAWST RF (0.842) and SAST RF (0.840) were marginally better than the MT RF, which obtained an *R*^2^ score of 0.826. The SAWST RF outperformed the SAWST LR model by 0.842 to 0.808, and similarly, the SAST and MT RF models outperformed their LR counterparts with scores of 0.840 to 0.810 and 0.826 to 0.810, respectively.

Discharges in Hospital 1 were more predictable than in Hospital 2. For instance, the *R*^2^ score for predicting discharges within the hour in Hospital 1 varied from 0.760 in LR models to 0.771 in RF models, whereas in Hospital 2, scores ranged from 0.626 to 0.684, respectively. Similarly, for predicting within the next 4 hours at Hospital 1, scores varied from 0.891 in LR models to 0.894 in RF models, whereas in Hospital 2, scores varied from 0.802 to 0.842, respectively. Predicting hourly discharges was also generally harder than predicting discharges within the next 4 hours. For Hospital 1, SAWST RF was the best-performing model for predicting discharges within 4 hours and obtained a score of 0.894 relative to 0.771 in the case of hourly discharges. For Hospital 2, SAWST RF was the best-performing model for predicting discharges within 4 hours and obtained a score of 0.842 relative to 0.684 in the case of hourly discharges.

**Table 2:** Performance profile for multitask and single task models for discharge within the next hour and discharges within the next 4 hours.

				Hospital 1							Hospital 2				
Metric				MT^a^		SAST^b^		SAWST^c^		MT			SAST		SAWST
**4 h MAE^d^, mean (SD)**															
	LR^e^			2.901 (0.12)		2.974 (0.15)		2.902 (0.12)		1.579 (0.12)			1.607 (0.11)		1.587 (0.12)
	RF^f^			2.850 (0.14)		2.847 (0.13)		2.834 (0.14)		1.485 (0.1)			1.456 (0.08)		1.437 (0.07)
**4 h *R*^2^, mean (SD)**															
	LR			0.891 (0.01)		0.887 (0.01)		0.891 (0.01)		0.810 (0.02)			0.802 (0.02)		0.808 (0.02)
	RF			0.893 (0.01)		0.894 (0.01)		0.894 (0.01)		0.826 (0.01)			0.840 (0.02)		0.842 (0.01)
**1 h MAE, mean (SD)**															
	LR			1.520 (0.04)		1.529 (0.05)		1.518 (0.04)		0.778 (0.05)			0.787 (0.05)		0.779 (0.05)
	RF			1.453 (0.05)		1.463 (0.04)		1.464 (0.04)		0.721 (0.04)			0.723 (0.04)		0.714 (0.04)
**1 h *R*^2^, mean (SD)**															
		LR	0.763 (0.01)		0.760 (0.01)		0.763 (0.01)		0.635 (0.03)			0.626 (0.03)		0.632 (0.03)	
		RF	0.770 (0.01)		0.771 (0.01)		0.771 (0.01)		0.668 (0.02)			0.679 (0.03)		0.684 (0.03)	

^a^MT: multitask.

^b^SAST: system-agnostic single-task.

^c^SAWST: system-aware single-task.

^d^MAE: mean absolute error.

^e^LR: linear regression.

^f^RF: random forest.

**Figure 3:**
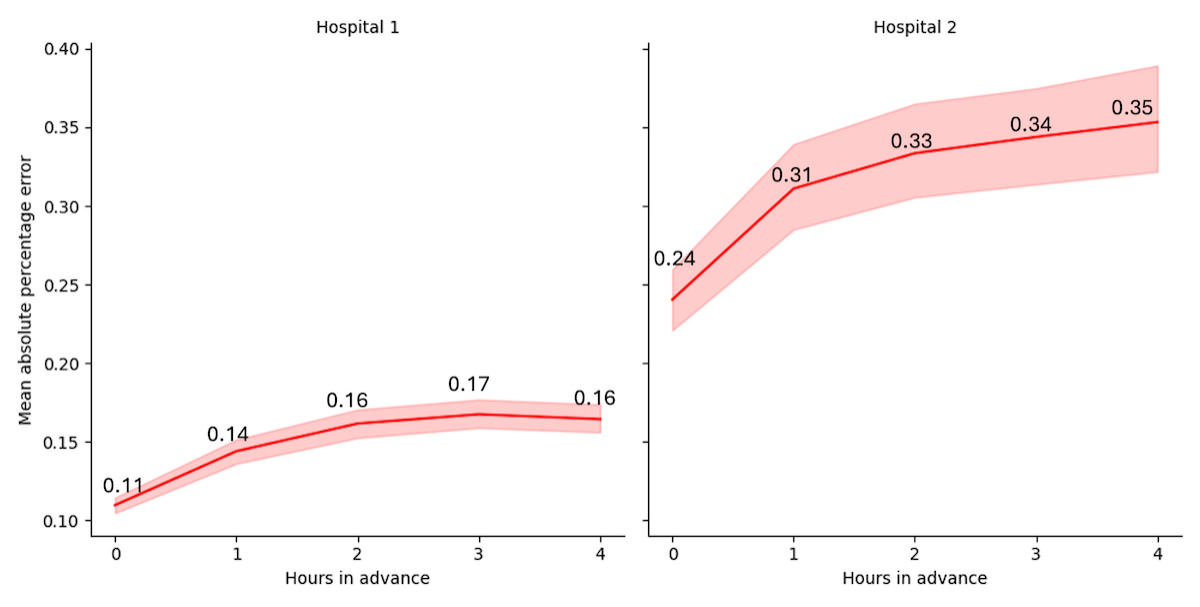
Mean absolute percentage error for predicting afternoon discharges as a function of the number of hours in advance.

The results of experiment 2 are summarized in Figure 3, where the x-axis corresponds to the number of hours in advance incurred to predict afternoon discharges, and the y-axis shows the MAPE with error bands with a 95% CI for the mean. These results are consistent with those from experiment 1, showing that predicting discharges at Hospital 2 is relatively harder than at Hospital 1. For Hospital 1, the MAPE varied from 0.11 to 0.16 when predicting from 0 to 4 hours in advance, respectively, representing a 45.45% performance decrease. In Hospital 2, the MAPE varied from 0.24 to 0.35, representing a 45.83% decrease. In addition, the model for Hospital 2 exhibited higher variance than the model for Hospital 1.

## Discussion

### Principal Findings

Our results show excellent performance when predicting hospital-level discharges within the next hour and the next 4 hours with and without access to external hospital data. We highlight that according to the MAE metric, the best model (ie, RF) for discharges within the next 4 hours has an average absolute error of 2.83 and 1.44 beds in hospitals 1 and 2, respectively. Relative to the 4-hour discharge average (ie, 20.80, SD 12.07 and 5.67, SD 4.93), these errors indicate reasonable predictive value with respect to overestimation or underestimation of the number of available beds in the next 1 to 4 hours. In both experiments, models developed for hospital 1 obtained better performance relative to hospital 2. This might be because hospital 1 is a large-scale academic trauma center, with highly standardized discharge workflows and more data available due to the size of the system. Hospital 2 is a smaller community hospital with less data available and more room to admit more variability in patient flow pathways.

Surprisingly, we found no evidence to suggest performance differences between the system-aware and system-agnostic models. When forecasting discharges in a network of hospitals where the facilities interact, variables from one hospital could be expected to impact discharges in the others. For instance, if some patients have clinical needs that are more aligned with a hospital different from where they initially arrived, transferring them to a different hospital would be preferred. However, if that hospital is at capacity, there might be a need to expedite discharges to accommodate an influx of incoming patients. Our two partner hospitals considered here indeed interact, but this interaction did not translate to improved performance in the context of system-aware models.

We hypothesize that the lack of performance increase in the context of system-aware models may be due to the discharge volume being more dependent on “within” hospital factors (ie, local variables) or because we failed to capture the “between” hospital variables that drive the interaction. This opens opportunities for future research to better understand the actual drivers of these interactions in the context of discharge predictions. Future research could explore other patient flow tasks that are less dependent on “within” hospital factors. For instance, predicting ED boarding time, which may depend on transfers to and from different hospitals, may still benefit from a system-aware approach.

Another potential explanation for the lack of difference in performance may be that the independent variables from both hospitals are correlated with each other (see Multimedia Appendix 1 for a correlation plot). For example, the number of discharge orders active in Hospital 1 are highly correlated with the discharge orders in Hospital 2 (correlation coefficient=0.75). To obtain a performance increase when considering a system-aware model, variables from the other hospitals in the system should bring additional information. However, since the independent variables considered in this study are of the same type for both hospitals and are correlated, adding such information does not seem to provide additional predictive value. Future work may consider additional variables such as the number of transfer orders originating from patients in location 1 that are being transferred to location 2 and vice versa. Such variables are hypothesized to bring additional value from a systemic perspective (ie, may enhance the predictive performance of system-aware models relative to system agnostic), but constructing and incorporating them into our main dataset is challenging due to the structure of the data stored in the EHR.

Another relevant finding is that predicting discharges in the next hour is generally more complicated than predicting discharges within the next 4 hours. This is likely because the coefficient of variation of the number of discharges in the next hour is higher than that of the number of discharges within the next 4 hours (see Table 1). This finding is relevant for practical purposes since decision-makers can use both types of forecasts for different purposes. For instance, the forecast of discharges in the next hour helps estimate the number of available beds in the short term and accommodate patients who need immediate admission. On the other hand, the forecasts of discharges within the next 4 hours can instead be used for longer-term planning in the ED and to accommodate patients who will eventually need to be admitted, but not immediately. Because of the lower performance, additional efforts may be required to operationally deploy the forecast of discharges in the next hour. Some efforts include adding independent variables, such as the ones described above.

Results from experiment 2 suggest that it is possible to accurately predict afternoon discharges up to 4 hours in advance using a simple set of independent variables like the one described in this study. Although the predictive performance decreases as the number of hours in advance from when the prediction is made, the model obtained good performance when predicting 4 hours in advance, particularly in hospital 1 (16% MAPE). The model’s performance for hospital 2 worsened (35% MAPE) when prediction took place 4 hours in advance. This finding is relevant because early prediction of afternoon discharges may help reduce ED boarding times, as resource availability can be identified earlier during the day, and this can help expedite patient admission workflows during the morning shift. Similarly, our models could indicate how to react to instances where we project low afternoon discharges and employ corrective actions to expedite discharges or plan alternative pathways for incoming inpatients.

Furthermore, in instances when there is capacity reserved for incoming surgical or ICU patients, a forecast of the number of expected discharges in the afternoon can serve to release some of the reserved capacity and admit ED patients early during the morning, knowing that there is a high possibility that there will be space available later during the day to accommodate incoming surgical or ICU patients. However, since prediction errors increase significantly as the forecast horizon extends, the models may have limited applicability when predicting four hours in advance, as these errors could result in surgical patients being stranded without an inpatient bed after their procedure. Similarly, the models could also be limited since we are assuming that there is a clear path between the information given by a prediction and the action that should be taken by decision makers using such predictions.

Unfortunately, although our predictions are informative, they are not prescriptive, and the value of the models will be limited by the decision maker’s acumen. Future research should explore advanced optimization techniques, such as constrained programming and reinforcement learning, that can help guide bed allocation decisions using such forecasts. With such techniques, instead of just providing a prediction directly to the users, we can provide a recommendation. This can be done by developing an optimization model that takes as input the predictions and outputs a recommended action, such as the number of beds currently allocated to surgical and ICU patients to free up for incoming ED patients.

### Implementation Considerations

A central insight of our study is that it showed that we can reliably make short-term discharge predictions using a relatively small number of independent variables. This can lead to broader adoption of such models since they do not require collecting, storing, or using an extensive set of variables. In addition, our approach’s temporal dynamic and aggregated nature, as opposed to the traditional static patient-level approach (eg, [12]), provides actionable insights that are updated as new information arrives. Our aggregated approach also yields a highly tractable framework that scales nicely with the number of hospitals. Although a dynamic patient-level approach might have advantages, it also has limitations. For example, in a network of hospitals with an average occupancy of 100 patients per day per facility, tracking every patient for 10 hours a day would require approximately 365,000 data points per year per facility. If interested in using 5 years of training data, that becomes 1.8 million records per hospital for training purposes. In hospitals with many facilities, this scales linearly with the number of facilities, but in increments that are in the order of 10^6^ per additional facility.

Although our approach comes with practical advantages, it also contains some potential limitations that can hinder the integration of our models into operational workflows. These include the use of retrospective data for model evaluation, technological limitations of the EHR system, the use of point predictions instead of prediction intervals, and the oversimplification of patient heterogeneity.

Unfortunately, not all the retrospective information used is readily available in real time. This is partly due to the different documentation processes across hospitals, where differences in workflows and data entry standards create delays or inconsistencies in how information is recorded. In addition, design limitations in medical records software further exacerbate this issue. For instance, retrospective data often resides in databases that are updated on a delayed schedule, such as every 24 hours, making it unsuitable for real-time decision-making. Meanwhile, real-time data is typically retrieved from separate systems that may not integrate seamlessly with the primary EHR platform used for retrospective analysis. This fragmentation between systems complicates efforts to translate retrospective insights into actionable real-time applications. Therefore, further work is needed to determine what subset of the independent variables this study considers can be used in real time.

Another limitation is that we only considered point predictions. A traditional stepping stone in the deployment of machine learning models is the issue of trust. A point prediction is generally not a trustworthy option to deploy a model, unless the model has a significantly small variance, and this is unlikely in most practical applications. Further efforts could be directed to using advanced techniques like quantile regression [25] to provide the decision maker with prediction intervals for which the prediction is expected to lie with a high degree of accuracy. These intervals would clearly be more informative than point prediction and could help to establish trust between the technology and its users.

Finally, hospital-level predictions may oversimplify patient heterogeneity. This can potentially lead to biased estimates if patient subgroups with distinct characteristics are not adequately represented in the aggregate data. To mitigate such biases, we propose the following as future directions: exploring hybrid models that combine aggregate and patient-level data and comparing the performance of models developed using aggregate versus patient-level data to quantify the potential impact of these biases if they exist.

### Conclusion

We developed and evaluated machine learning models to predict short-term discharges in multiple health care systems. We evaluated models with and without access to systemic data (ie, data from other hospitals) and found no difference between the two information tiers. We found that accurate forecasts can be made using a simple set of explanatory variables, even when predicting discharge hours in advance.

## Appendix

Multimedia Appendix 1 Additional study details.

## Abbreviations

ED: emergency department
EHR: electronic health record
GCB: general care bed
ICU: intensive care unit
IRB: institutional review board
IU: inpatient unit
LR: linear regression
MAE: mean absolute error
MAPE: mean absolute percentage error
MT: multitask
RF: random forest
SAST: system-agnostic single-task
SAWST: system-aware single-task
