# Correction: Toward Real-Time Discharge Volume Predictions in Multisite Health Care Systems: Longitudinal Observational Study

**DOI:** 10.2196/83802

**Published:** 2025-09-23

**Authors:** Fernando Acosta-Perez, Justin Boutilier, Gabriel Zayas-Caban, Sabrina Adelaine, Frank Liao, Brian Patterson

**Affiliations:** 1 Department of Industrial and Systems Engineering University of Wisconsin–Madison Madison, WI United States; 2 UW Health: University of Wisconsin Hospitals and Clinics Madison, WI United States; 3 Department of Emergency Medicine University of Wisconsin–Madison Madison, WI United States

In “Toward Real-Time Discharge Volume Predictions in Multisite Health Care Systems: Longitudinal Observational Study” (J Med Internet Res 2025;27:e63765) the authors noted several errors in the references and added one new reference.

The following selections of the text were published with incorrect references:

“Although few studies have explored the use of multitask learning [18] for patient flow [15,16]...”

“The literature has considered 2 main types of models: patient-level models [7-11] and hospital-level models [12,13]. Hospital-level models typically use a time series approach to directly predict the number of discharges in an entire hospital or a hospital unit such as the ED [12,13].”

“...while others treat the discharge prediction problem as a classification task and predict the risk that a patient will be discharged in the next 24 to 48 hours [7-9].”

“We note that patient-level predictions can also be used to estimate discharge volume by aggregating the patient-level discharge probability estimates [7-9].”

“We used two models: linear regression (LR) with L1 regularization [20,21] and a random forest (RF) [19,22].”

These reference citations have been updated to link them to the correct references in the list, and a new reference has been added (number [22] in the updated list). Consequently, references 18-24 have been renumbered.

The correction will appear in the online version of the paper on the JMIR Publications website, together with the publication of this correction notice. Because this was made after submission to PubMed, PubMed Central, and other full-text repositories, the corrected article has also been resubmitted to those repositories.
