# Supplementary material for: Toward Real-Time Discharge Volume Predictions in Multisite Health Care Systems: Longitudinal Observational Study
Source: J Med Internet Res. 2025 Apr 30;27:e63765. doi: 10.2196/63765 (PMC12079059; doi:10.2196/63765)
Supplement: Multimedia Appendix 1 [file jmir_v27i1e63765_app1.docx]

Supplemental Materials

# Complete List of Independent Variables

In table 1, we carefully describe every independent variable used in our study. Except the temporal variables, all other variables were computed for both hospitals. There is a total of 60 variables per hospital. Therefore, in the system aware and multitask setting, there was a total of 120 variables. For all variables we included lag terms, which captured the history of a particular variable for the previous six hourly periods. For example, for a row in our dataset corresponding to 12:00 PM, we included the number of discharges one period ago (i.e., from 11:00 AM to 12:00 PM), two periods ago (from 10:00 AM to 11:00 AM) and so on until six periods ago. This same procedure was repeated for all other variables in the patient movement, medical orders, and time in bed types.

Table 1: Independent variables descriptions and abbreviations

| Variable Type | Abbreviation | Description |
| --- | --- | --- |
| Temporal | Hour | Hour |
|  | Hour-SQ | Hour Squared |
|  | Day_Number | Day of the month |
|  | day_of_year | Day of the year |
|  | week_of_year | Week of the year |
|  | month | Month |
|  | day-hour | Observation number |
|  | Monday | Sunday |
|  | Saturday | Monday |
|  | Sunday | Tuesday |
|  | Thursday | Wednesday |
|  | Tuesday | Thursday |
|  | Wednesday | Saturday |
| Patient Movement | Discharges-1 | Number of discharges one period ago |
|  | Discharges-2 | Number of discharges two periods ago |
|  | Discharges-3 | Number of discharges three periods ago |
|  | Discharges-4 | Number of discharges four periods ago |
|  | Discharges-5 | Number of discharges five periods ago |
|  | Discharges-6 | Number of discharges six periods ago |
|  | Transfers-1 | Number of net transfers one period ago |
|  | Transfers-2 | Number of net transfers two periods ago |
|  | Transfers-3 | Number of net transfers three periods ago |
|  | Transfers-4 | Number of net transfers four periods ago |
|  | Transfers-5 | Number of net transfers five periods ago |
|  | Transfers-6 | Number of net transfers six periods ago |
| Medical Orders | Orders | Number of discharge orders active now |
|  | Orders-1 | Number of discharge orders one period ago |
|  | Orders-2 | Number of discharge orders two period ago |
|  | Orders-3 | Number of discharge orders three periods ago |
|  | Orders-4 | Number of discharge orders four periods ago |
|  | Orders-5 | Number of discharge orders five periods ago |
|  | Orders-6 | Number of discharge orders six periods ago |
| Time in Bed | TIB24 | Number of patients with time in bed  ≤ 24 now |
|  | TIB24-1 | Number of patients with time in bed  ≤ 24 one period ago |
|  | TIB24-2 | Number of patients with time in bed  ≤ 24 two periods ago |
|  | TIB24-3 | Number of patients with time in bed  ≤ 24 three periods ago |
|  | TIB24-4 | Number of patients with time in bed  ≤ 24 four periods ago |
|  | TIB24-5 | Number of patients with time in bed  ≤ 24 five periods ago |
|  | TIB24-6 | Number of patients with time in bed  ≤ 24 six periods ago |
|  | TIB63 | Number of patients with time in bed  >24 and ≤ 63 now |
|  | TIB63-1 | Number of patients with time in bed ≤r 24 and <50 one period ago |
|  | TIB63-2 | Number of patients with time in bed  >24 and ≤ 63 two periods ago |
|  | TIB63-3 | Number of patients with time in bed  >24 and ≤ 63 three periods ago |
|  | TIB63-4 | Number of patients with time in bed  >24 and ≤ 63 four periods ago |
|  | TIB63-5 | Number of patients with time in bed  >24 and ≤ 63 five periods ago |
|  | TIB63-6 | Number of patients with time in bed  >24 and ≤ 63 six periods ago |
|  | TIB148 | Number of patients with time in bed  >63 and ≤ 148 now |
|  | TIB148-1 | Number of patients with time in bed  >63 and ≤ 148 one period ago |
|  | TIB148-2 | Number of patients with time in bed  >63 and ≤ 148 two periods ago |
|  | TIB148-3 | Number of patients with time in bed  >63 and ≤ 148 three periods ago |
|  | TIB148-4 | Number of patients with time in bed  >63 and ≤ 148 four periods ago |
|  | TIB148-5 | Number of patients with time in bed  >63 and ≤ 148 five periods ago |
|  | TIB148-6 | Number of patients with time in bed  >63 and ≤ 148 six periods ago |
|  | TIB148+ | Number of patients with time in bed  ≥ 148 now |
|  | TIB148+-1 | Number of patients with time in bed  ≥ 148 one period ago |
|  | TIB148+-2 | Number of patients with time in bed  ≥ 148 two periods ago |
|  | TIB148+-3 | Number of patients with time in bed  ≥ 148 three periods ago |
|  | TIB148+-4 | Number of patients with time in bed  ≥ 148 four periods ago |
|  | TIB148+-5 | Number of patients with time in bed  ≥ 148 five periods ago |
|  | TIB148+-6 | Number of patients with time in bed  ≥ 148 six periods ago |

# Model Selection and Parameter Tuning

We determined the hyper-parameters of the models using the training data and a K-Fold cross validation approach. The number of estimators in the random forest was decided adhoc to be 200. In experiment 1, we tuned the models independently for every information tier and each hospital: system aware, system agnostic, and multitask. For linear regression, we evaluated values of the coefficient of regularization that varied from 0.001 to 0.50 in increments of 0.002. For the decision tree, we evaluated the max depth of the tree from 1 to 20. In table 2 we present the hyper-parameters used in experiment 1. In both parts of experiment 2, system aware random forest model with 200 estimators was used for all evaluations.

Table 2: Hyper-parameters used in experiment 1

| Model | Location | Target Type | Parameters |
| --- | --- | --- | --- |
| Random Forest | 1  2 | Hourly  Four Hours  Hourly  Four Hours | number of estimators=200 number of estimators=200 number of estimators=200 number of estimators=200 number of estimators=200 number of estimators=200 number of estimators=200 number of estimators=200 |
|  |  | All | number of estimators=200 |
| Linear Regression | 1  2 | Hourly  Four Hours  Hourly  Four Hours | L1 Coefficient=0.001  L1 Coefficient=0.001  L1 Coefficient=0.001  L1 Coefficient=0.001  L1 Coefficient=0.001  L1 Coefficient=0.001  L1 Coefficient=0.001  L1 Coefficient=0.001 |

# Cross Validation Visualization

In the main text, we described in words the cross validation procedure. See Figure 1 for a visual representation of such procedure.


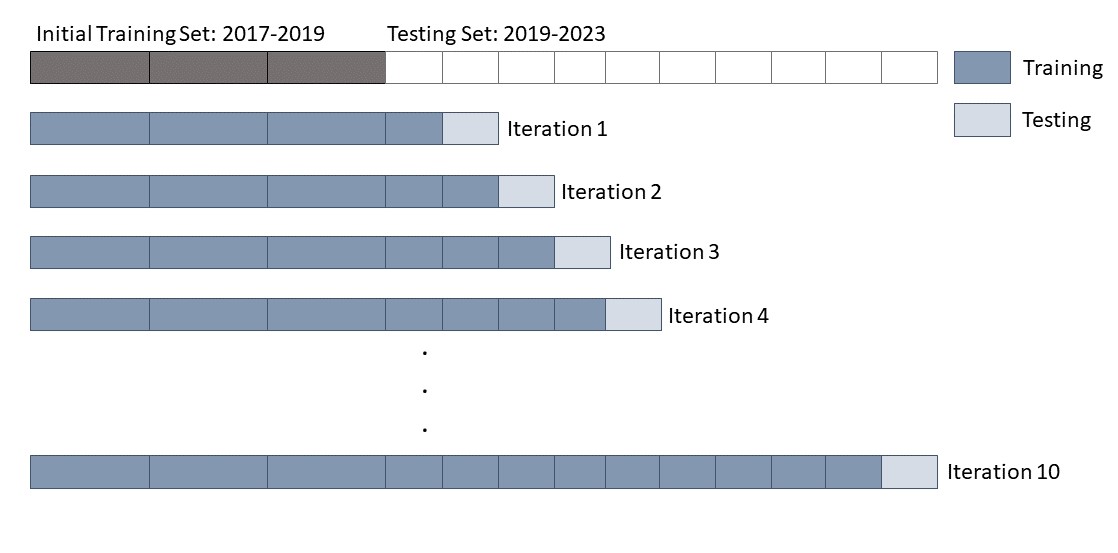


Figure 1: Conceptualization of the cross-validation procedure.

# Feature Importance Results for Hourly and Four Hour Discharges

4.1 General Feature Importance for Experiment 1

To get a sense of what features were important, we trained a linear regression with an L1 coefficient of regularization of 0.001 at each location only with data available at that location (i.e., system agnostic). Once the model was trained, we explore the coefficients of the linear regression model to estimate feature importance. In the context of linear regression small negative numbers (large in magnitude) and large positive numbers are considered important. In figure 1, we sorted the coefficients from biggest to smallest and plotted the first five (with positive coefficients) and last five (with negative coefficients). For every location and target (hourly or four hours), discharge orders at the time of prediction were one of the most important features. Other features that were consistently identified as important are: previous discharges, hour of the day, and different variations of features related to time in bed. Refer to table 1 for a description of the variable abbreviation. Interestingly, hours squared obtained a negative coefficient. This might be since when looking at the number of discharges as a function of the hour of the day, there is a concave relationship, since discharges are low during early morning, peak close to noon, and then are low again in the afternoon. The hour squared with a negative coefficient captures this.

4.2 Relationship Between Discharge Orders and Actual Discharges

A significant amount of the prediction performance comes from the variables related to discharge orders. In Figure 3, we show the relationship between the number of discharge orders at the time of predictions (x-axis) and the number of discharges in the next hour and next four hours (y-axis). As it can be seen in the figure, there is a strong linear relationship between the number of discharges active and the number of discharges in the next four hours. In the hourly setting, this correlation is subject to a greater degree of dispersion. We believe that this is part of the reason of why hourly discharges are harder to predict.

# Correlation Analysis

The correlation heat map, we only include non-lag components. When comparing the correlation of the variables of hospital 1 with variables of hospital 2, one can note that the greatest correlation comes from the order variables and the previous discharge variables. For instance, the orders variable in location 1 (i.e., L1-Orders), exhibits a 0.75 correlation with orders in location 2 (i.e., L2-Orders). Similarly, previous discharges in location 1 (i.e., L1-Discharges-1) have a a correlation score of 0.67 with previous discharges in location 2 (i.e., L2-Discharges-1).


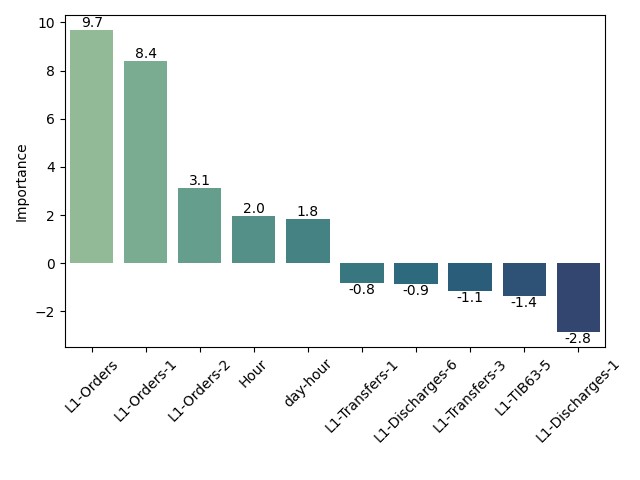

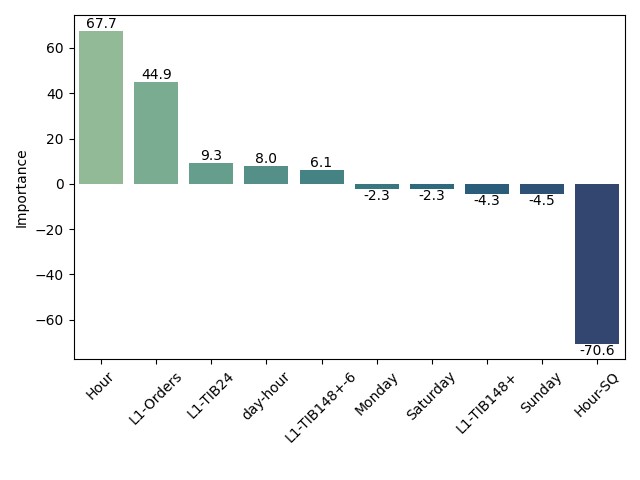


(a) Hourly model in L1 (b) Four hour model in L1


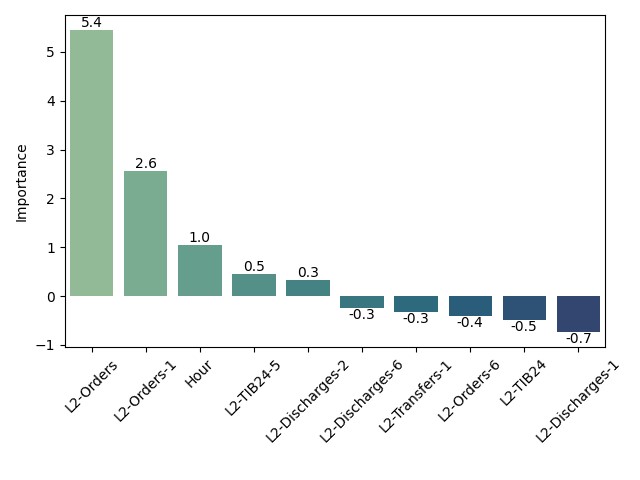

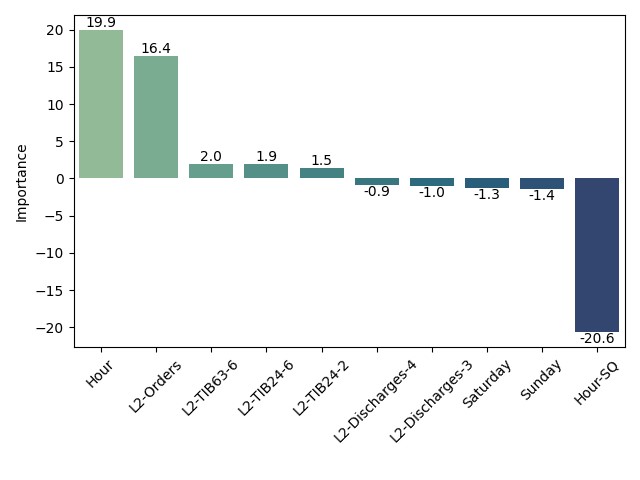


(c) Hourly model in L2 (d) Four hour model in L2

Figure 2: Feature importance results for system agnostic models in both locations (L1= Location 1, L2= Location 2)

# Difference Between Early Morning and Afternoon Performance

In Figure 5, we present the different performance between early morning discharges, and afternoon discharges. We used data from experiment one for such assessment. We categorized as "AM" as the time points when predictions were made before 12:00 PM, and "PM" otherwise. The models obtained a higher error when predicting early morning discharges. Our models rely in data from previous periods (i.e., lag variables) to make discharges predictions. However, early mornings are a period of low hospital activity (i.e., not much is happening between 12:00 AM and 8:00 AM). Thus, in early morning there is not much information that can be leveraged to predict discharges.

On the other hand, when predicting after 12:00 PM, discharges can be predicted more accurately using the rich information available from the daily operations of our partner hospital systems. We highlight that the fact that morning discharges are more difficult to predict is not a discouraging finding. In fact, operationally, we care more about the afternoon discharges, since this is is typically the time of high demand of inpatient resources coming from different sources such as the ED and the operating room.

# Experiment 2 Model Training Details

Although we only evaluated the model in the period of interest (12:00 PM to 4:00 PM), the models were trained to predict four hour discharges with one to four hours in advance in all


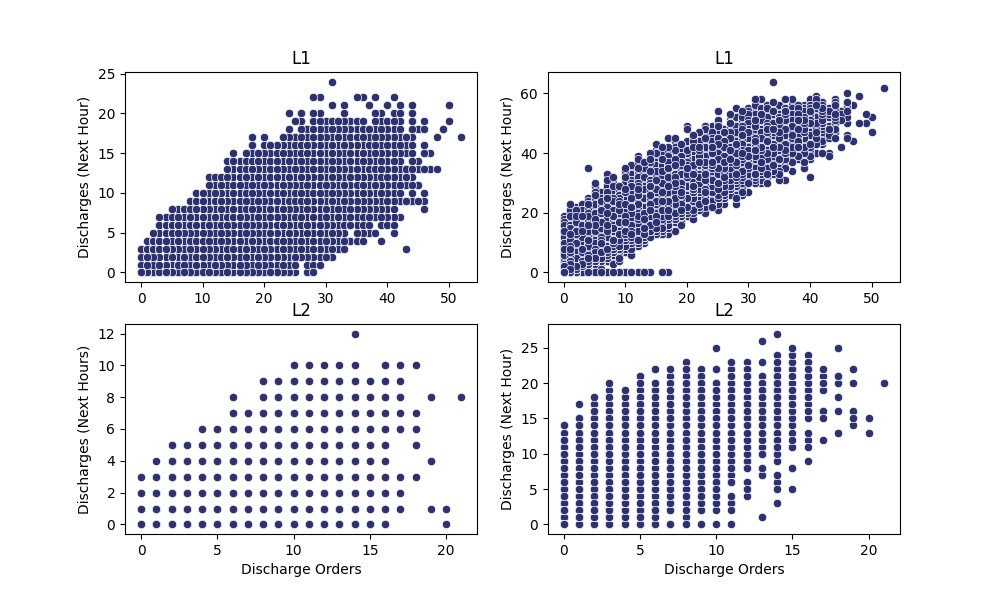


Figure 3: Relationship between discharge orders and discharges in the next hour and next four hours


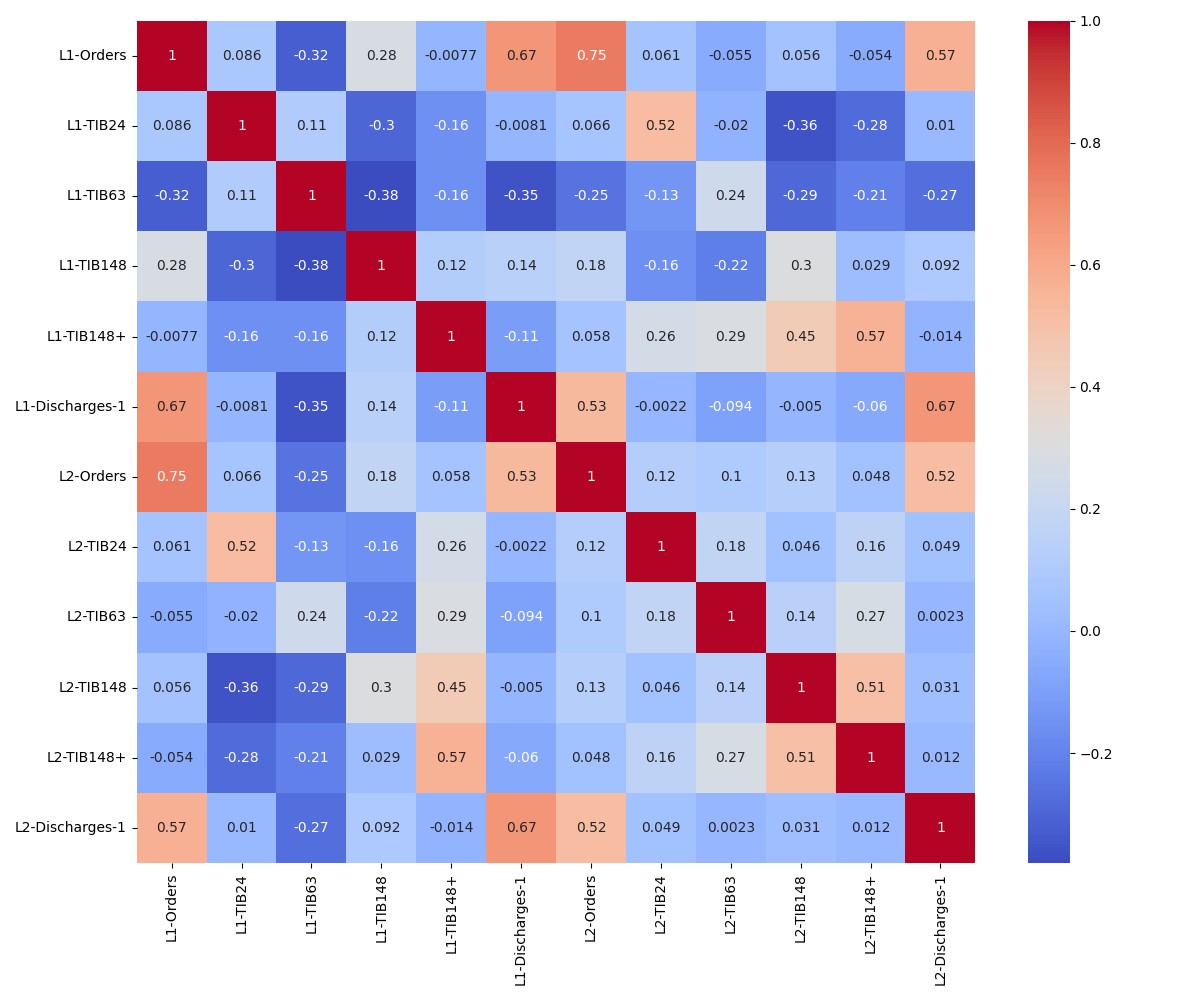


Figure 4: Correlation heat map for the main independent variables included in the study.

four hour periods starting from 8:00 AM to 4:00 PM. To understand the training procedure for the models in experiment 2, recall that every row in our dataset represents an hour of the day. As an example, consider the period of 8:00 AM. When predicting one hour in advance, the prediction represents the number of discharges from 9:00 AM to 1:00 PM. Since our dataset contains all the time periods from 8:00 AM to 5:00 PM, this prediction procedure was repeated for all periods from 8:00 AM to 5:00 PM, but we only care about the instance at which we are predicting the time window of 12:00 PM to 4:00 PM, which in the case of one hour corresponds to the period of 11:00 AM. We trained five separate models (i.e., one for each numbers of hour in advance), and then evaluated the performance on the time window of 12:00 PM to 4:00 PM for each model. The rationale behind this architecture is to train a model that "learns" patterns from predicting discharges in other periods and exploits that knowledge when predicting discharges in the period of interest (i.e., 12:00 PM to 4:00 PM).


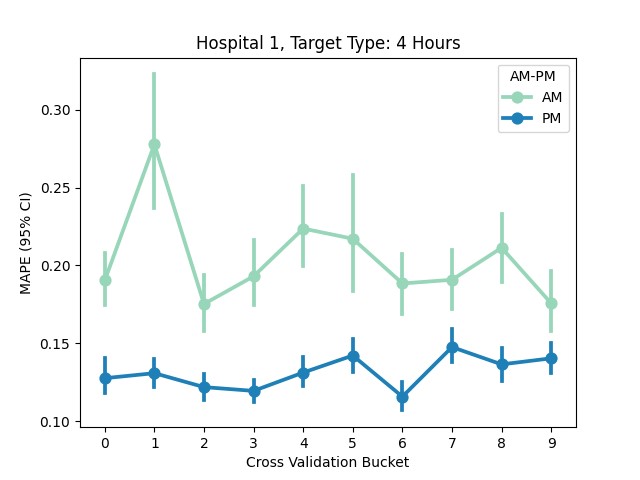

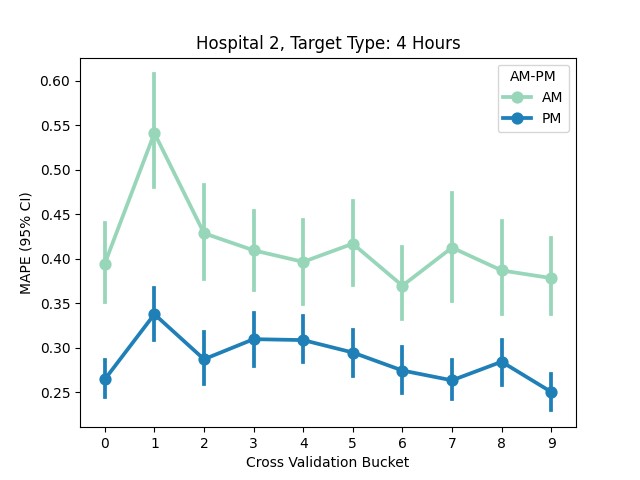


Figure 5: Mean absolute error for predicting discharges within the next four hours.
